# Development of Inhalable Spray Dried Nitrofurantoin Formulations for the Treatment of Emphysema

**DOI:** 10.3390/pharmaceutics15010146

**Published:** 2022-12-31

**Authors:** Mathew N. Leslie, Nirmal Marasini, Zara Sheikh, Paul M. Young, Daniela Traini, Hui Xin Ong

**Affiliations:** 1 Respiratory Technology, The Woolcock Institute of Medical Research, Glebe, Sydney, NSW 2037, Australia; 2 Faculty of Medicine, Healthy and Human Sciences, Macquarie University, Sydney, NSW 2109, Australia; 3 School of Pharmacy, Brac University, Dhaka 1212, Bangladesh; 4 Department of Marketing, Macquarie Business School, Macquarie University, Sydney, NSW 2109, Australia

**Keywords:** nitrofurantoin, spray-dried, emphysema, two-fluid nozzle, three-fluid nozzle, extracellular matrix

## Abstract

A central characteristic of emphysematous progression is the continuous destruction of the lung extracellular matrix (ECM). Current treatments for emphysema have only addressed symptoms rather than preventing or reversing the loss of lung ECM. Nitrofurantoin (NF) is an antibiotic that has the potential to induce lung fibrosis as a side effect upon oral administration. Our study aims to repurpose NF as an inhalable therapeutic strategy to upregulate ECM expression, thereby reversing the disease progression within the emphysematous lung. Spray-dried (SD) formulations of NF were prepared in conjunction with a two-fluid nozzle (2FN) and three-fluid nozzle (3FN) using hydroxypropyl methylcellulose (HPMC) and NF at 1:1 *w*/*w*. The formulations were characterized for their physicochemical properties (particle size, morphology, solid-state characteristics, aerodynamic behaviour, and dissolution properties) and characterized in vitro with efficacy studies on human lung fibroblasts. The 2FN formulation displayed a mass mean aerodynamic diameter (MMAD) of 1.8 ± 0.05 µm and fine particle fraction (FPF) of 87.4 ± 2.8% with significantly greater deposition predicted in the lower lung region compared to the 3FN formulation (MMAD: 4.4 ± 0.4 µm; FPF: 40 ± 5.8%). Furthermore, drug dissolution studies showed that NF released from the 2FN formulation after 3 h was significantly higher (55.7%) as compared to the 3FN formulation (42.4%). Importantly, efficacy studies in human lung fibroblasts showed that the 2FN formulation induced significantly enhanced ECM protein expression levels of periostin and Type IV Collagen (203.2% and 84.2% increase, respectively) compared to untreated cells, while 3FN formulations induced only a 172.5% increase in periostin and a 38.1% increase in type IV collagen. In conclusion, our study highlights the influence of nozzle choice in inhalable spray-dried formulations and supports the feasibility of using SD NF prepared using 2FN as a potential inhalable therapeutic agent to upregulate ECM protein production.

## 1. Introduction

Chronic obstructive pulmonary disease (COPD) is an umbrella term for diseases such as emphysema and chronic bronchitis, which collectively account for 212.3 million cases and 3.3 million deaths reported globally, standing as the 3rd highest cause of mortality worldwide [1]. Emphysema is characterized by the progressive and irreversible loss of the lung extracellular matrix (ECM). Lung ECM comprises a complex and dynamic structure of fibrous proteins (collagen, elastin), glycoproteins (fibronectin, laminin, tenacin), glycosaminoglycans (heparin, hyaluronic acid) and proteoglycans (perlecan, versican) that play a fundamental role in maintaining lung homeostasis, in addition to providing structural integrity [2]. The human lung is continuously exposed to inhaled microbes, allergens, particulate matter and smoke and thus undergoes a continuous process of repair and regeneration via the organization of existing ECM molecules and the production of new ECM molecules. Therefore, the loss of ECM in the emphysematous lung alters the macromolecular network of proteins that comprise the ECM matrix, resulting in reduced lung function and increased lung susceptibility to the damages caused by external stimuli [3]. Currently, there are no treatments available for halting or reversing the progressive ECM destruction associated with emphysema [4].

Nitrofurantoin (NF), a broad-spectrum antibiotic indicated for prophylactic use in urinary tract infections (UTI), has been shown to induce lung fibrosis after long-term use [5,6,7]. The upregulation of ECM production during fibrosis suggests that NF could potentially be repurposed to utilise this fibrotic effect as a novel means of treating the emphysematous lung. The antibiotic nature of nitrofurantoin may also serve as a beneficial secondary effect, as respiratory infections are a frequent source of exacerbations in emphysema [8,9]. Although NF presents potential benefits, its application is restricted by low aqueous solubility and suboptimal pharmacokinetic profiles including a short plasma half-life (<1 h) and large urinary elimination (40–50%) following oral administration [10]. It was found that a 100 mg oral dose of nitrofurantoin resulted in a blood concentration of less than 1 µg/mL [11], thus limiting the use of the drug for the treatment of local bacterial infections via the oral route. In this context, delivery of NF directly to the lungs by inhaled formulations could overcome the pharmacokinetic limitations of NF by using lower doses, thereby reducing the dose-related toxic effects of the drug. Furthermore, it is expected that an inhaled NF may provide a targeted therapy with localised interaction with lung fibroblasts, a cell type that is responsible for ECM maintenance, potentially reversing the damage caused during emphysema.

Spray drying is a widely employed particle-engineering technique that can produce fine particles for inhaled therapies. This technique is also a cost-effective method of producing highly consistent particles that can be delivered through a dry powder inhaler (DPI) and are environmentally friendly as the use of a propellant is not required. Spray drying uses an aqueous or organic/aqueous solution/suspension of the desired drug, alone or in combination with a carrier, to be fed into the spray dryer and atomised into droplets by a suitable nozzle before being dried by currents of heated air. Several factors, including feed flow rate, inlet and outlet temperature, aqueous/organic contents, drug/carrier physicochemical properties and nozzle size impact the final characteristics of the spray-dried powder [12]. In particular, the type of nozzle used to atomise the feed (drug solution/suspension) plays a significant role in the patterns of drug distribution in the dried particles [12,13,14,15]. This is especially significant as the drug distribution within the particles then dictates the drug dissolution characteristics [14]. A variety of atomising nozzles are used in spray drying, such as rotary, hydraulic (one-fluid), ultrasonic and pneumatic (two-fluid or three-fluid) nozzles [12,13]. The particles produced by each type of nozzle will display varying drug distribution and physicochemical characteristics due to the different methods of atomisation employed by each nozzle type. Furthermore, the differences in particle characteristics due to nozzle type allow for greater control over the qualities of the resulting drug formulation. The two-fluid nozzle (2FN) is the most commonly used pneumatic nozzle and functions by ejecting a stream of the drug/carrier solution from a single channel. This method of spray drying produces uniformly dispersed drugs within and on the particle surface [14,16,17]. Conversely, a three-fluid nozzle/3FN offers distinct advantages in terms of spraying patterns as both the drug/carrier solution can be separated into aqueous and organic phases that are simultaneously sprayed from an inner and an outer channel. The separation of the inner and outer channels results in the encapsulation of inner channel droplets by outer channel droplets, enabling particles in which an active pharmaceutical ingredient (API) is encapsulated within an excipient to improve the pharmacokinetic properties of the drug [18]. Therefore, the selection of nozzle type is an important determinant in developing an inhaled formulation as it allows for the optimisation of drug formulations to produce robust inhalable therapies.

The study aims to develop an inhalable spray-dried formulation of NF and hydroxypropyl methylcellulose (HPMC) as a polymeric carrier using the traditional 2FN nozzle and compares it with a spray-dried NF formulation produced by the 3FN nozzle as potential treatments for emphysema. HPMC was chosen as the excipient in these two formulations as the polymer is inert, biocompatible and well-studied [19,20,21,22]. Furthermore, HPMC has been found to prolong drug release and improve aerosolization of inhaled therapies [20]. The physicochemical characterisations (particle size, thermal properties and release profiles) and aerosolisation behaviour of the two formulations were compared. Finally, the efficacy of these formulations was further tested in vitro on a lung fibroblast cell line to determine whether the formulations could upregulate ECM protein production.

## 2. Materials and Methods

### 2.1. Materials

Nitrofurantoin, hydroxypropyl methylcellulose (HPMC, viscosity 80–120 cp, 2% in H_2_O), acetone, and Hanks’ Balanced Salt Solution (HBSS) were purchased from Sigma Aldrich (Sydney, Australia). The solvent acetonitrile was obtained from Chem-Supply Pty Ltd. (Adelaide, Australia). Phosphate buffered saline (PBS) was purchased from Gibco, Invitrogen (Melbourne, Australia). Water was purified by reverse osmosis (MilliQ, Millipore, Guyancourt, France). All solvents used were of analytical grade. 

### 2.2. Preparation of Spray-Dried Nitrofurantoin Formulations

The nitrofurantoin-based spray-dried formulations were prepared using a Büchi mini spray dryer B-290 (Büchi Laboratories, Flawil, Switzerland). Dry powder nitrofurantoin and HPMC were prepared at a 1:1 ratio (*w*/*w*) and dissolved in acetone and Milli Q water, respectively. The solution of nitrofurantoin and acetone was sonicated for 5 min by an Ultrasonic Cleaner (Shanghai Bilon Instrument Co., Shanghai, China) to ensure complete dissolution. The dry powder HPMC and Milli Q water solution were heated to 80 °C and stirred for 1 h to ensure the polymer was fully solubilised. The two solutions were then either mixed at a 1:4 ratio (*v/v*) before being sprayed by a two-fluid nozzle (2FN) or individually fed into the spray dryer and sprayed from a three-fluid nozzle (3FN). The spray-dried formulation prepared using a two-fluid nozzle and three-fluid nozzle are denoted as 2FN and 3FN, respectively. Additionally, a formulation denoted as spray-dried NF was prepared using identical settings to 2FN excluding the addition of HPMC. 

#### 2.2.1. Two-Fluid Nozzle

Nitrofurantoin (0.8 g) was added to 200 mL of acetone while HPMC (0.8 g) was separately added to Milli Q water (800 mL). The nitrofurantoin-acetone solution was then diluted (1:4 *v/v*) by the HPMC-Milli Q water solution to achieve 0.16% *w/v*. This percentage was chosen to ensure that the poor solubility of nitrofurantoin could be accommodated while minimising precipitation. The spray drying conditions used with the 2FN were as follows: inlet temperature of 140 °C, outlet temperature of 90 °C, feed rate of 3.15 mL/min, aspiration 100% (35 m^3^/h), and atomiser setting at 40 mm (~540 L/h).

#### 2.2.2. Three-Fluid Nozzle

Nitrofurantoin (0.8 g) was added to acetone (200 mL) while 0.8 g of HPMC was separately dissolved in 800 mL of Milli Q water. The two solutions were then separately fed into a 3FN. The HPMC solution (1 mg/mL) was sprayed from the outer channel and the nitrofurantoin solution (4 mg/mL) was sprayed from the inner channel of the 3FN. The two solutions together thus had a collective final feed concentration of 0.16% *w/v*. The spray drying conditions used for the 3FN formulation were identical to that of the 2FN formulation excluding the type of nozzle used and feed pump setting as 3FN requires two liquid feeds. 3FN formulation was produced using the following feed pump settings: inner pump feed rate of 0.63 mL/min of the organic 4 mg/mL nitrofurantoin solution, outer pump feed rate of 2.52 mL/min for the aqueous 1 mg/mL HPMC in Milli Q water solution.

### 2.3. Quantification of Nitrofurantoin Using High-Performance Liquid Chromatography

Nitrofurantoin content was assessed using a high-performance liquid chromatography (HPLC) system (Shimadzu, Kyoto, Japan) according to a validated method [23]. The HPLC system was equipped with an SPD-20A UV–Vis detector, LC20HT pump and SIL20AHT autosampler (Shimadzu, Japan). A mobile phase consisting of acetone and Milli Q water was prepared in a 20:80 ratio (*v/v*) with a potassium phosphate trihydrate buffer (25 mM) and pH adjusted to pH 3 using >85% phosphoric acid (*v/v*; Sigma-Aldrich). Samples were diluted as required using a mixture of acetone and Milli Q water (20:80 (*v/v*) ratio) and analysed at a wavelength of 366 nm, at a flow rate of 1 mL/min with an injection volume of 100 µL using a Luna C18, 100A column (Phenomenex, Los Angeles, CA, USA), 150 × 4.6 mm. Linearity was obtained between 0.01–50 µg/mL (R^2^ = 0.995) with a limit of detection (LoD) of 0.001 µg/mL and a limit of quantification (LoQ) of 0.005 µg/mL.

### 2.4. Scanning Electron Microscopy

Spray-dried powder morphology was examined using JEOL JCM-6000 scanning electron microscopy (JCM-6000PLUS Neoscope, JEOL, Tokyo, Japan). Approximately 5 mg of each sample was placed onto carbon tape mounted onto an aluminium stub and gold-coated by a sputter coater (Smart coater, JEOL, Japan) for 2 min, resulting in a ~20 nm coating thickness. Images were taken at a voltage of 15 kV using a range of magnifications.

### 2.5. Particle Size Analysis

The particle size distribution of spray-dried powders and the raw drug was analysed by laser diffraction using a Mastersizer 3000 system (Malvern Instruments, Malvern, UK) equipped with a dry powder dispersion system (Malvern Aero S.). Approximately 30 mg of powder was analysed at an obscuration between 0.1–15% and dispersed by 3 bars of compressed air pressure. Particle size distribution corresponding to the 10th, 50th and 90th percentile of particles in the respective formulations was represented as the D10, D50 and D90, respectively. The particle population dispersity determined by the span value was calculated using the following formula:

Span value = (D90 − D10)/D50(1)

### 2.6. Powder Tapped Density

Powder density was determined in triplicate using a previously reported method [14]. The powder equivalent of 1 g of sample was placed in a 5 mL measuring cylinder and the volume was recorded as the bulk volume. The measuring cylinder was tapped on a flat bench at approximately 100 taps/min until the sample volume remained consistent and the volume was recorded as the tapped volume. Bulk and tapped densities of the formulations were then determined by dividing the mass of the powder by the volume before and after tapping. 

### 2.7. In Vitro Aerodynamic Performance

The aerosol deposition profiles of the spray-dried NF formulations were determined using a next-generation impactor (NGI, Apparatus X, Copley, Nottingham, UK) fitted with a 90° USP induction port (representing the throat) as specified in the United States Pharmacopeia (USP). To minimise particle bounce, the throat and the NGI stages were coated with a mixture of Brij 35 (1 g), ethanol (4 mL), and glycerol (5 mL). Briefly, the NGI was connected to a high-capacity vacuum pump and the airflow rate was set to 60 L/min using a calibrated flowmeter (TSI 4040; TSI Instruments Ltd., Shoreview, MN, USA). A size 3 gelatine capsule (Capsugel, Morristown, NJ, USA) filled with approximately 20 mg of spray-dried powder was loaded into low-resistance dry powder inhalers (DPI; RS01, Plastiape, Osnago, Italy). A DPI device was attached to the USP induction port (throat) via a 3D-printed mouthpiece adaptor. The gelatine capsule was pierced and actuated for 4 s which corresponded to one breath. Following aerosolisation, the drug powders deposited in the device, capsule, NGI stages (1–7) and micro-orifice collector (MOC) were collected by rinsing with a mixture of acetonitrile and water (ACN: H_2_O) at a 20:80 (*v/v*) ratio and quantified using HPLC. All samples were diluted with ACN: H_2_O (20:80) at a 1:10 ratio and filtered using syringe filters (PTFE, 0.45 µm) before analysis with HPLC. Aerodynamic particle size distribution (APSD) was calculated using Copley Inhaler Testing Data Analysis Software (CITDAS) version 3.1 (Copley, Nottingham, UK) to calculate the fine particle fractions (FPF; the ratio of emitted dose below 5 µm against the total delivered dose), the geometric standard deviation (GSD) and the mass median aerodynamic diameter (MMAD) and characterize the aerosol performance of the 2FN and 3FN formulations.

### 2.8. Dissolution of Nitrofurantoin

Dissolution profiles for 2FN and 3FN formulations were assessed in triplicates using a Franz diffusion cell equipped with magnetic stirrers and heated jackets (V6B, permeGear Inc., Bethlehem, PA, USA). Spray-dried powders (6 mg) were evenly dispersed on a nitrocellulose membrane (0.45 µm, MF membrane filters, Millipore Bedford, MA, USA) and secured between the apical and basal compartments of each cell. Each cell was filled with dissolution media (22.7 mL of 7.4 pH PBS) and maintained at 37 ± 0.5 °C by a circulating peristaltic pump (Carter-Manostat, Pumpworks Inc., Sanford, FL, USA) set to 5 L/min and stirred at a constant rate by magnetic stirrer bars. This system was heated to 37 ± 0.5 °C for at least 30 min to equilibrate before the experiment. Samples (0.5 mL) were taken from the receiver compartment at pre-determined time points and replaced with an identical volume of preheated PBS (0.5 mL) over 3 h. At the end of the experiment, the membrane was washed on the apical side to determine the content of undissolved NF, diluted in a 1:10 ratio (*v/v*) with a mixture of acetone and Milli Q water (20:80 *v/v*) and quantified using HPLC. The similarity factor (*f*2), difference factor (*f*1) and dissolution modelling were calculated using the Microsoft Excel add-in “DDSolver” [24]. *f*2 values between 50 and 100 are considered equivalence or sameness between two profiles, respectively, whereas *f*1 values between 15–100 indicate a difference between dissolution profiles [25,26].

### 2.9. X-ray Diffraction

The X-ray diffraction (XRD) patterns of nitrofurantoin, HPMC, SD nitrofurantoin, SD HPMC, 2FN and 3FN were assessed using a Panalytical Aeris (Malvern Panalytical Ltd., Malvern, UK) at room temperature. A 15 kV cobalt X-ray source with a current of 40 mA was used to determine the diffraction patterns with an angular increment of 0.022 °C/s over a diffraction angle of 2θ in the range of 5–40 °C.

### 2.10. Dynamic Vapour Sorption

The capacity of the powders for moisture sorption was determined through dynamic vapour sorption (DVS) (DVS-1 Intrinsic, Surface Measurement Systems Ltd., London, UK) with baseline isothermal conditions at 25 °C with 0% relative humidity (RH). Powders (approximately 10 mg) were exposed to two sorption and desorption cycles of 0–90% RH at an increment of 10% then returned to 0% RH in 10% increments with each cycle repeated twice. The mass of powders was measured at each 10% RH increment of both cycles. RH was kept constant until the drug mass was consistent enough for the sample moisture content change per minute (dm/dt) to remain below 0.01%/min with a minimum time of 10 min for each RH value. This delayed method of measurement was used to determine the equilibrium moisture content for each step.

### 2.11. Differential Scanning Calorimetry

Differential Scanning Calorimetry (DSC; Mettler-Toledo DSC823e, Schwerzenbach, Switzerland) was used to examine the thermal properties of the spray-dried formulations. Samples (5 mg) were sealed in aluminium pans. The pan was pierced and heated at 10 °C/min ranging from 25–440 °C with an empty aluminium pan as a reference sample. A flow controller was connected to maintain a nitrogenous environment with approximately 30 mbar within the system (dry gas) and approximately 10–20 mbar in the oven (purge gas). DSC data were analysed by the STARe software version 11.00a (Mettler-Toledo, Scherzenbach, Switzerland).

### 2.12. Karl Fischer Titrator

The moisture content of the powders was determined using Karl Fischer titration (KFT; DL31, Mettler-Toledo, Scherzenbach, Switzerland). A sample (approximately 5 mg) was placed in the vessel and iodine was gradually added until all moisture in the sample had been eliminated by an oxidation reaction. The volume of eliminated iodine was used to calculate the percentage moisture content of each sample.

### 2.13. Thermogravimetric Analysis

Thermogravimetric analysis (TGA, Mettler-Toledo) was performed to examine the thermal stability of the spray-dried formulations. The mass of the powder formulations (~5 mg) was analysed over a range of 25–800 °C at a heating rate of 10 °C/min with a nitrogen flow rate of 25 mbar. Data was analysed through the STARe software version 11.00a (build 4393, Mettler-Toledo, Schwerzenbach, Switzerland).

### 2.14. In Vitro Bio-Characterisation

#### 2.14.1. Cell Culture

MRC-5 cells (healthy human lung fibroblast cell line) and NCI-H441 (alveolar epithelial cell line) were purchased from the American Type Cell Culture Collection (ATCC, Manassas, VA, USA). MRC-5 cells were maintained in modified eagle medium (MEM; Gibco), supplemented with 10% (*v/v*) foetal bovine serum (FBS; Invitrogen), 1% (*v/v*) sodium pyruvate (Gibco), 7.5% (*v/v*) sodium bicarbonate (Gibco) and 1% (*v/v*) non-essential amino acids (Sigma-Aldrich, Australia) then incubated at 37 °C with CO_2_. MRC-5 cells were seeded in 24-well plates (Corning Costar, Cambridge, MA, USA) at a density of 6 × 10^4^ cells/cm^2^. 

NCI-H441 cells were maintained in RPMI 1640 media (Gibco) supplemented with 10% (*v/v*) FBS (Invitrogen) at 37 °C with 5% CO_2_ (*v/v*). NCI-H441 cells were seeded at a density of 3 × 10^5^/cm^2^. All experiments were conducted between a population doubling level of 28–42 for the MRC-5 cell line and between passage numbers 45–78 for the NCI-H441 cell line.

#### 2.14.2. Air-Liquid Interface (ALI) Culture of NCI-H441 Cells

To create an air-liquid interface (ALI) model of NCI-H441 cells, Transwell cell culture inserts (0.33 cm^2^, polyethylene terephthalate (PET) membrane, 0.4 µm pore size) (Corning Costar) were used as previously described [27]. Briefly, NCI-H441 cells were seeded at a density of 9 × 10^4^/cm^2^ in RPMI 1640 media supplemented with 10% (*v/v*) FBS separately on the apical compartment of a Transwell membrane and the same media was added to the basolateral chamber. The cells were incubated at 37 °C with 5% CO_2_ for 24 h. To initiate ALI conditions for the NCI-H441 cells, media in the apical chamber was removed after 24 h and the cells were maintained under ALI conditions for 2 weeks. Differentiation media in the basolateral chamber was replaced every 3 days. All experiments were performed 2 weeks post-ALI induction as three biological replicates.

#### 2.14.3. Drug Transport Study

Drug transport study for 2FN and 3FN formulations was conducted across the ALI cultures of NCI-H441 cell line over 6 h. Using a method previously presented [28], 2FN and 3FN spray-dried formulations were suspended in a model propellant, 2H,3H-Decafluoropentane (HPFP; Apollo Scientific Ltd., Stockport, UK) (0.2 mg/mL), and then the suspensions (50 µL) were added to the apical chamber, allowing the propellant to evaporate, leaving a layer of consistent drug deposition on the apical, chamber with HBSS (600 µL) was placed at the basolateral chamber. Samples (200 µL) were taken from the basolateral chamber every 30 min for the first 2 h and then every hour for the final 4 h with samples being replaced by fresh, warmed HBSS. After 6 h, the apical chamber was washed twice with HBSS to collect any remaining drug with a pipette tip (denoted as On). To quantify the amount of drug present inside the cells, the cell layer was then scraped from the Transwell insert membrane by adding HBSS and centrifuged at 13,000× *g* for 10 min. The resulting supernatant was discarded, and the cell pellet was resuspended in cell-Lytic^TM^ (Invitrogen) buffer and passed through a 26 G needle 8–10 times to lyse the cells. All the samples were then analysed using HPLC.

#### 2.14.4. Sodium Fluorescein Permeability Assay

Epithelial barrier integrity of the ALI cultures before and after the drug transport study was assessed via sodium fluorescein permeability assay (Na-Flu) to determine whether the drug deposition altered the integrity of the ALI cultures. Briefly, sodium fluorescein (2.5 mg/mL), (Sigma Aldrich) was added to the apical chamber of untreated cells (control) and on treated cells following drug deposition and 6 h transport study while pre-warmed HBSS was added to the basolateral chamber. After the transwells were incubated for 6 h at 37 °C with 5% CO_2_, basolateral samples (200 µL) were collected at 0, 15, 30 and 60 min to measure the rate of transport (flux) of the sodium fluorescein from the apical chamber to the basolateral chamber. For analysis, the collected basolateral samples were diluted (1:20) and fluorescence was measured using the SpectraMax M2 plate reader (excitation: 485 nm; emission: 538 nm). The apparent permeability (Papp) of Flu-Na through NCI-H441 cells through the following formula:

(Papp) = (dQ)/(dT × C_0_ × A) (2)

where dQ/dT represents the flux of sodium fluorescein (µg/s) across the membrane, C_0_ is the initial donor concentration (µg/mL) and A is the surface area (cm^2^).

#### 2.14.5. Efficacy Study Using ECM ELISA

Enzyme-linked immunosorbent assay (ELISA) for ECM proteins was performed to investigate the efficacy of 2FN and 3FN as a potential treatment for emphysema according to a previously described method [29]. 24 h prior to the experiment, MRC-5 cells were seeded in 24-well plates (Corning Costar) that had been coated with 10 µg/cm^2^ of rat tail collagen-1 (Sigma Aldrich) at a density of 9.5 k cells/cm^2^ in supplemented MEM media. The Transwell inserts containing NCI-H441 ALIs were placed on top of each well containing a monolayer of MRC-5 cells at the bottom. Then, 50 μL of the 2FN or 3FN suspension in HPFP (0.2 mg/mL) was added to the Transwell apical chamber, allowed to dry and incubated at 37 °C with 5% CO_2_ to allow the transport of NF to occur. The ALIs were removed post 6 h of treatment and the cells were incubated at 37 °C with 5% CO_2_ for a further 72 h. Once treatment was complete, the cells were washed twice with phosphate-buffered saline (PBS) and decellularized using 16 mM NH_4_OH before further PBS washing. The decellularized ECM was then treated with BSA (0.1%, Sigma Aldrich) to block nonspecific binding and treated with monoclonal mouse anti-perlecan primary antibody (diluted 1:2000; Invitrogen), monoclonal rabbit anti-collagen-IV (diluted 1:2000; Sigma Aldrich), monoclonal mouse anti periostin primary antibody (diluted 1:2000, BD Biosciences, Franklin Lakes, NJ, USA), monoclonal mouse anti-fibronectin primary antibody (diluted 1:4000, Invitrogen) or a monoclonal mouse anti tenacin-C primary antibody (diluted 1:8000, Sigma Aldrich) and subsequently an HRP-linked anti-mouse secondary antibody (diluted 1:2000; Cell Signalling Technology, Danvers, MA, USA). The ECM was then treated with an HRP-linked anti-rabbit secondary antibody (diluted 1:2000; Cell Signalling Technology) followed by absorbance measurement using the SpectraMax M2 plate reader (excitation: 450 nm; emission: 570 nm).

### 2.15. Statistical Analysis

All results are expressed as mean ± standard error of the mean of at least 3 independent replicates. The statistical software, GraphPad Prism (version 8.2.1, San Diego, CA, USA) was used to test for significance by performing unpaired *t*-test, One-Way or Two-Way ANOVA for each experiment. Significance was determined as *p* < 0.05.

## 3. Results

### 3.1. Preparation of Spray-Dried Powder Formulations

Nitrofurantoin was spray-dried with the excipient HPMC at a 1:1 *w*/*w* ratio using either a 2FN or a 3FN. The spray dryer settings, powder yield and final feed concentration were kept consistent between the 2FN and 3FN spray drying. The nozzle type used for each formulation was found to affect a range of powder properties, including total powder yield, moisture content, and density (Table 1). The final yield of powder produced by 2FN was higher compared to 3FN, with significantly lower moisture content compared to 3FN (*p* < 0.05, Table 1). Furthermore, 2FN powders showed significantly lower bulk and tapped density compared to 3FN powders (*p* < 0.05, Table 1). Both formulations also had a lower proportion of nitrofurantoin compared to the theoretical 1:1 ratio (50%), however, the drug content of 2FN was found to be significantly higher than 3FN.

### 3.2. Physico-Chemical Characterisation

#### 3.2.1. Scanning Electron Microscopy

**Figure 1:**
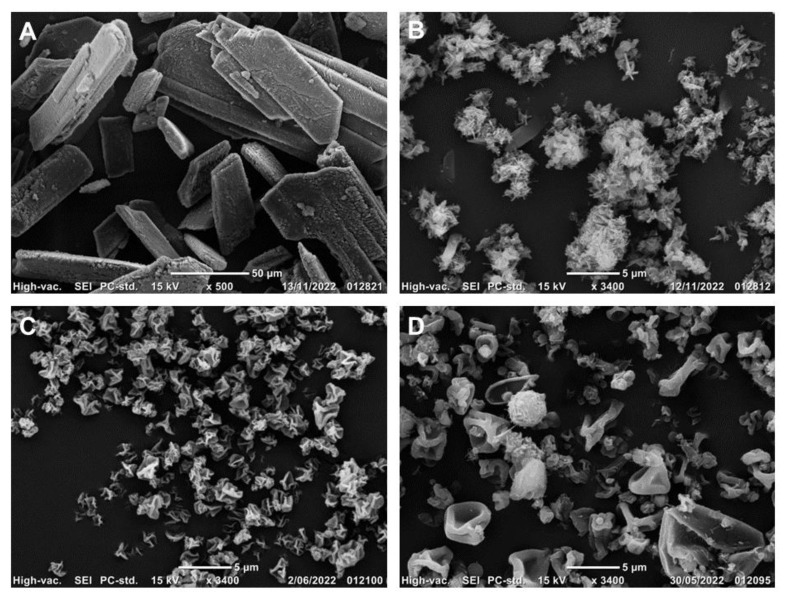
Scanning electron microscopy images displaying: (**A**) Raw nitrofurantoin (Magnification × 500); (**B**) Spray-dried nitrofurantoin (without HPMC; ×3400); (**C**) 2FN (×3400) and (**D**) 3FN (×3400).

Representative images of raw nitrofurantoin and the spray-dried NF powders are shown in Figure 1. Raw NF showed irregular elongated rod-shaped structures whereas spray-dried NF (raw drug only) displayed a diverse yet smaller particle size with rough crystalline surface morphology. 2FN powder exhibited monodispersed highly corrugated particles while 3FN powder displayed corrugated and comparatively diverse morphological patterns. The morphology of the spray-dried NF was indicative that the formulation was unsuccessful without the polymeric carrier HPMC, thus this formulation was not a focus of this study.

#### 3.2.2. X-ray Diffraction

**Figure 2:**
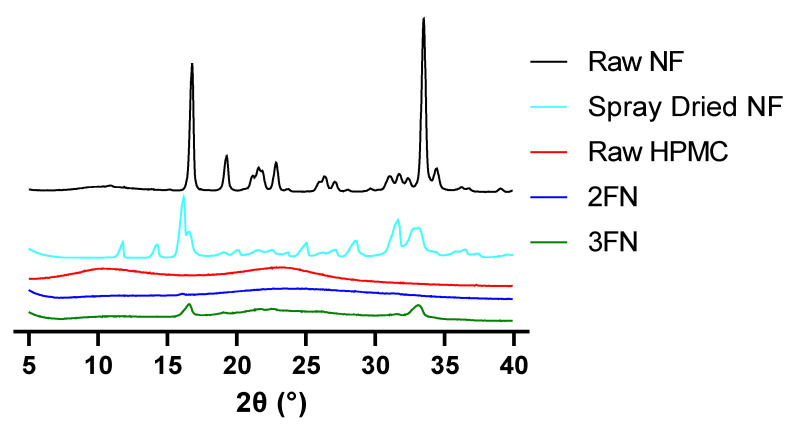
X-ray diffraction patterns produced by 2FN and 3FN formulations along with raw NF, spray-dried NF (drug only) and raw HPMC.

X-ray diffraction patterns of raw NF, spray-dried NF, raw HPMC, 2FN and 3FN are displayed in Figure 2. Raw nitrofurantoin and spray-dried nitrofurantoin are highly crystalline, with high-intensity peaks at 16.8 and 33.5° and many lesser crystalline peaks also detected between 18.8 and 32.5°. The crystalline peaks of NF were of lower intensity in the spray-dried NF and further suppressed by the addition of the excipient HPMC. Two peaks were detected at 16.6 and 33.1° for 3FN indicating partial crystallinity, however, no such peaks were observed for 2FN, thus indicating the successful formation of an amorphous structure for this formulation. 

#### 3.2.3. Particle Size

The size distribution of the powders measured by laser diffraction is shown in Table 2. Both formulations showed a larger proportion of the powder with an aerodynamic diameter of less than <5 µm, suggesting that the formulations are within the respirable fraction. The 2FN powders showed significantly lower particle size than 3FN (Table 2). The D50 representing 50% of the particle size population for 2FN was found to be significantly lower compared to 3FN (Table 2). Importantly, 2FN showed a significantly lower D90 (4.24 ± 1.88 μm) in contrast to that of 3FN (*p* < 0.01, Table 2). 2FN powders were distributed monomodally (span value 2.86) compared to 3FN (span value 3.16); however, no significant differences were observed between them (Table 2).

### 3.3. Thermal Analysis of Nitrofurantoin

#### 3.3.1. Differential Scanning Calorimetry and Thermogravimetric Analysis

**Figure 3:**
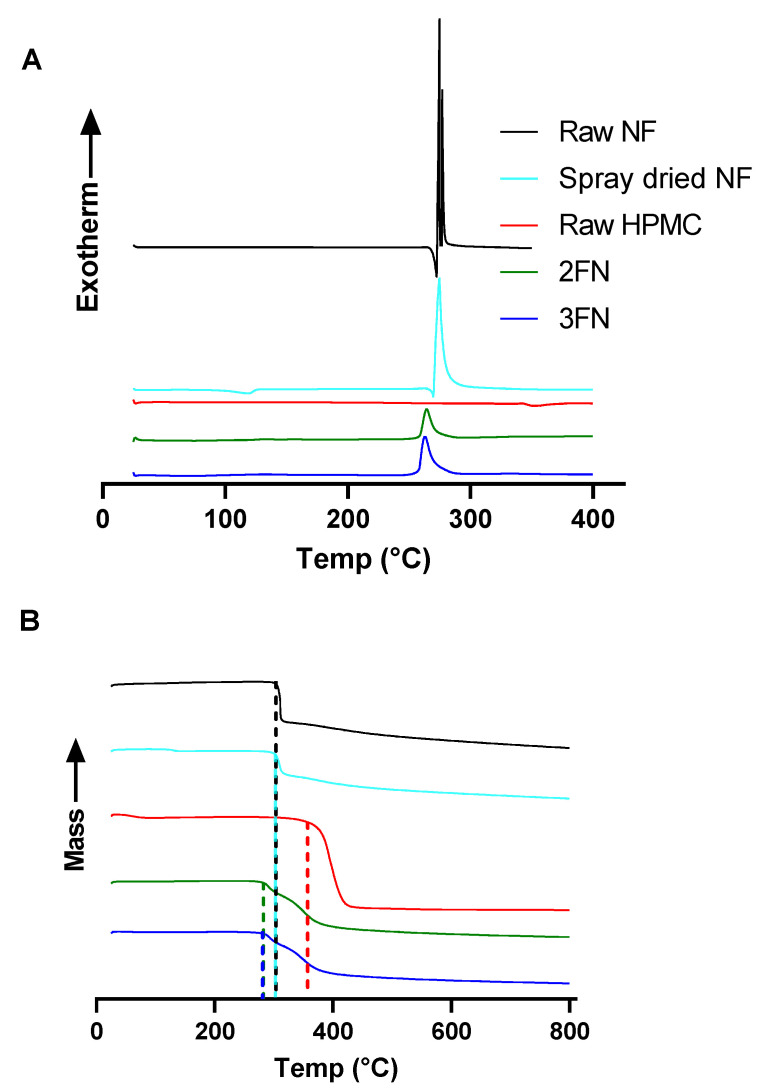
(**A**) Differential scanning calorimetry thermograms displaying the thermal properties of 2FN, 3FN powders, raw NF, spray dried NF and raw HPMC. (**B**) Thermogravimetric Analysis of 2FN, 3FN, raw NF, raw HPMC and spray-dried NF.

The thermal properties of 2FN and 3FN were determined using DSC and TGA (Figure 3). HPMC showed a broad and shallow endothermic peak from 350 to 360 °C, indicating an amorphous nature after spray-drying (Figure 3A). Raw NF displayed a short endothermic peak at 272 °C as the melting point of nitrofurantoin was reached and then two sharp adjacent exothermic peaks occurred at 277 °C and 280 °C, as the thermal degradation of nitrofurantoin begins. Similarly, spray-dried NF alone displayed an endothermic peak at 269 °C and a single exothermic peak at 274 °C due to thermal degradation. Both 2FN and 3FN thermograms showed only a single exothermic peak at 264 °C, possibly due to the amorphous nature of the particles. 

Thermogravimetric analysis displayed the change in mass that occurs across a range of temperatures (Figure 3B). When heated from 0–800 °C, mass sharply dropped at 309 °C for raw NF and spray-dried NF. However, for the raw HPMC, the mass gradually dropped to 343 °C. Both 2FN and 3FN powders displayed a similar pattern of change in mass at 256 °C due to the addition of HPMC to the spray-dried formulation.

#### 3.3.2. Dynamic Vapor Sorption

**Figure 4:**
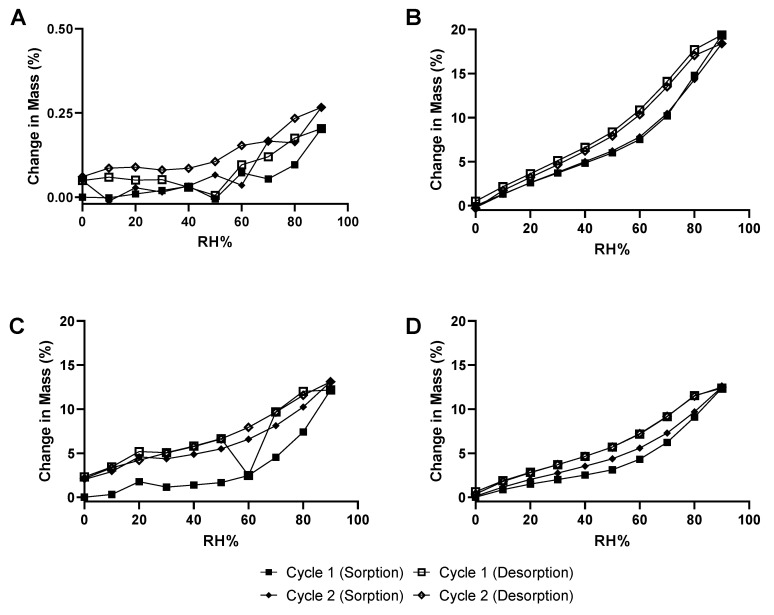
Moisture sorption isotherms of (**A**) Raw nitrofurantoin (**B**) Raw HPMC (**C**) 2FN and (**D**) 3FN. The percentage change in mass at 25 °C is shown when RH % was increased from 0–90% (sorption) and 90–0% (desorption) twice, represented as cycle 1 and cycle 2.

The moisture sorption profiles of raw nitrofurantoin, raw HPMC, 2FN and 3FN were examined to determine the effect of changes in relative humidity (RH%) on powder properties (Figure 4). Raw NF (Figure 4A) showed an approximate 0.3% change in mass at 90% RH, whereas the raw HPMC (Figure 4B) displayed 18.4% change in mass. All powders displayed the greatest % change in mass at 90% RH. The spray-dried formulations showed a maximum % change in mass of 13.1 and 12.5% for 2FN and 3FN, respectively. Notably, the moisture sorption profiles of raw HPMC and 3FN are reversible and showed a similar pattern of steadily increasing mass due to RH, yet raw HPMC adsorbed water vapour at a greater rate than 3FN. Conversely, raw nitrofurantoin and 2FN did not display reversible sorption profiles, as raw nitrofurantoin mass remained negligible until 60% RH was reached then rose to a maximum of 0.3%. However, the sorption profile of 2FN was found to be reversible after the first sorption cycle was completed, likely due to the elimination of residual solvents. The change in mass of 2FN was observed to drop sharply to approximately 2.5% at 60% RH during the first desorption cycle before immediately rising back to a 7% change in mass.

### 3.4. Nitrofurantoin Deposition Profile

**Figure 5:**
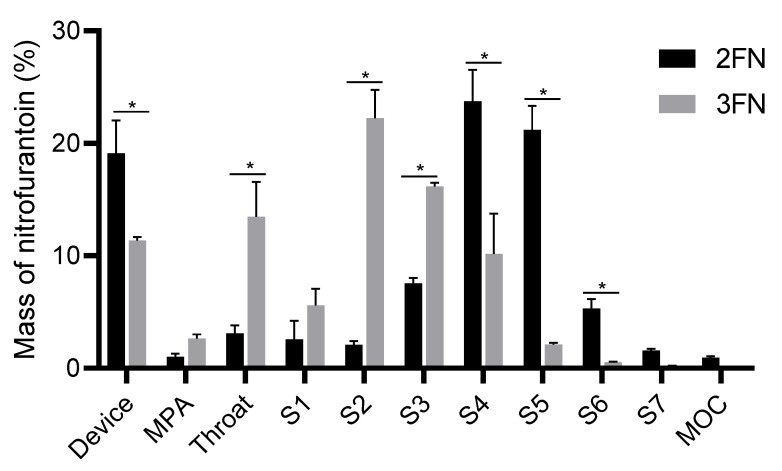
Next-generation impactor stage deposition profiles of 2FN and 3FN formulations. (Device = Dry Powder Inhaler, MPA = mouth-piece adaptor, MOC = micro-orifice collector). Data are reported as mean ± SD, *n* = 3, * *p* < 0.05.

The in vitro aerosolization performance of the formulations was assessed using the NGI (Figure 5, Table 3) and the spray-drier nozzle type was found to greatly impact the aerosol performance of the powders. In the case of 2FN formulation, the deposition of powder was primarily in the later stages (S3–S6) while for 3FN formulation, the deposition was in the early to mid-stages of the NGI (Throat-S4). 2FN displayed a significantly higher FPF (87.4 ± 2.8%) compared to 3FN (40.0 ± 5.8%). Furthermore, 2FN was found to have a significantly lower MMAD (1.78 ± 0.1 μm) than 3FN (4.36 ± 0.4 μm). However, the GSD values of the two powders were similar (Table 3). Additionally, the emitted dose (mg) was similar between the formulations (2FN: 6.90 ± 0.78 mg, and 3FN: 7.32 ± 0.12 mg, Table 3), with no significant differences between them. Notably, the high device retention was related to residual moisture in the formulations that had not been eliminated during spray drying. Overall, 2FN showed better aerosol performance compared to 3FN.

### 3.5. Dissolution Profile

**Figure 6:**
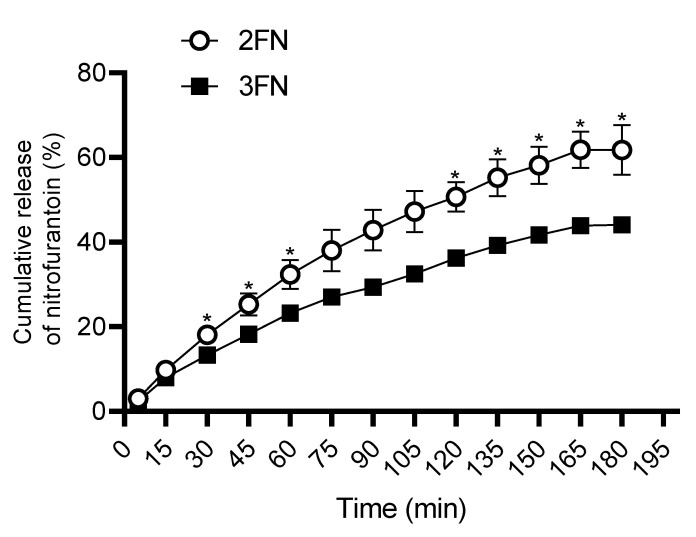
Dissolution profiles of nitrofurantoin from 2FN and 3FN over 3 h. The cumulative release of nitrofurantoin (%) was sampled in 15 min intervals to a maximum of 180 min and quantified by HPLC. Data are expressed as Mean ± SD, *n* = 3, * *p* < 0.05.

Drug release from 2FN and 3FN was determined over three hours using the Franz-diffusion cell (Figure 6, Table 4). The NF release pattern of 2FN was significantly higher compared to 3FN between 30 and 60 min and from 120 min until the experiment was completed. Approximately, 60% and 40% of NF were released within 3 h by 2FN and 3FN, respectively, demonstrating that the choice of nozzle produced powders of differing dissolution profiles despite the similarities in preparation. The difference factor (*f*1) was calculated to compare the dissolution profiles of the formulations. The *f*1 of the dissolution profiles was determined to be 23.91, indicating that the two dissolution curves are likely to be different as an *f*1 value ranging between 15 and 100 is considered equivalent. However, the formulations showed a borderline similarity factor (*f*2 = 50.7), where 50–100 is considered similar. The kinetics of the drug release pattern was modelled for various equations and both powders were found to be most accurately modelled by Korsmeyer-Peppas ‘Power Law’ drug release model (2FN: R^2^= 98.82%, 3FN: R^2^ = 99.16%). The release exponent (‘n’ value) of 2FN and 3FN when modelled by the Korsmeyer-Peppas model was 0.636 and 0.661, respectively, suggesting that drug release from the both formulations is governed by anomalous (non-Fickian) transport [30].

### 3.6. Drug Transport

**Figure 7:**
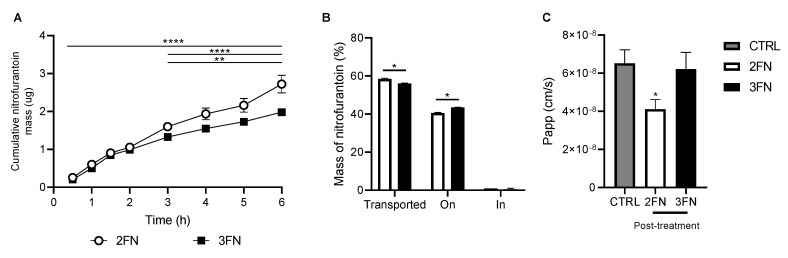
Transport of 2FN and 3FN spray-dried formulations. (**A**). Cumulative mass of nitrofurantoin transported across H441 ALI cultures for 2FN and 3FN formulations. (**B**). Percentage of the total mass of NF transported (shown as Transported), remaining on the apical layer (On) and inside the cells (In) at the end of 6h transport study (**C**). Comparison of apparent permeability (Papp) of ALI cultures for 2FN and 3FN formulations post-treatment (6 h) with untreated cells (control). (*n* = 3, mean ± SD) (* *p* < 0.05, ** *p* < 0.01, **** *p* < 0.0001) using two-way ANOVA with Tukey’s posthoc test).

A drug transport study was performed to investigate whether 2FN and 3FN could cross the epithelial layers of an alveolar epithelial cell line (H441) over a 6 h period. The cumulative mass of NF increased over time during the 6 h transport study for both 2FN and 3FN (Figure 7A). No significant difference in cumulative mass transported was found between the 2 powder formulations from the start until 2 h. After 3 h, a significant increase in cumulative mass transported was observed for 2FN compared to 3FN (*p* < 0.001, Figure 7A). The cumulative mass transported after 6 h was significantly higher for 2FN compared to 3FN (Figure 7A). At the end of the 6h transport study, a negligible amount of nitrofurantoin was found inside the cells (shown as ‘In’, Figure 7B) whereas the percentage of drug remaining on the apical layer of the cells was 40.6% for 2FN and 43.4% for 3FN (Figure 7B).

To determine whether drug deposition during the 6 h transport study affected the epithelial barrier integrity of the cellular layers, a sodium fluorescein (Na-Flu) permeability assay was conducted before and after the transport study. 2FN formulation showed a significant decrease in Na-Flu Papp value (4.1 × 10^−8^ cm/s,) while 3FN showed no significant difference compared to the control (untreated cells) (Figure 7C). These results indicate that 2FN increased the tightness of the epithelium while 3FN showed no change in epithelial barrier integrity, suggesting that both formulations did not negatively impact the H441 cell barrier functions. The increase in epithelial tightness due to 2FN did not translate to a reduction in 2FN transport compared to that of 3FN. Overall, the results show that both 2FN and 3FN formulations were transported across the alveolar epithelial cells to reach the target site. 

### 3.7. 2FN and 3FN Induce ECM Production

**Figure 8:**
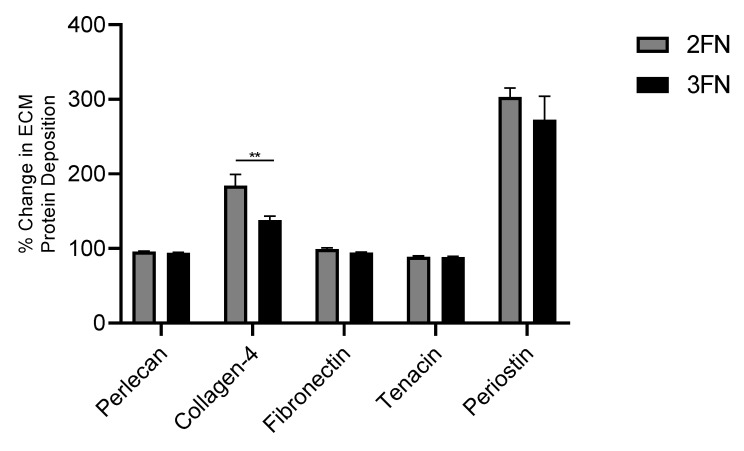
Percentage change in healthy fibroblast (MRC-5) ECM protein deposition when treated with 2FN or 3FN. Data are displayed as mean ± SD, *n* = 3, ** *p* < 0.01.

An ECM Enzyme-Linked Immunosorbent Assay (ELISA) was conducted to investigate whether 2FN and 3FN formulations induced ECM production in healthy MRC-5 cells (Figure 8). No significant increase in ECM production was observed for perlecan, fibronectin or tenacin-C by 2FN or 3FN when compared against control cells. However, a significant increase in both periostin and collagen-IV production was observed for cells treated with 2FN or 3FN. 2FN showed a significant increase in collagen-IV production compared to 3FN, indicating the better efficacy of 2FN compared to 3FN.

## 4. Discussion

Lung fibroblasts are known to reduce ECM maintenance during emphysema, leading to progressive lung damage. Therefore, for the first time, the present study aimed to repurpose the fibrotic side effect of NF by upregulating ECM production in emphysematous lung fibroblasts and restoring the structural integrity of the lungs. In our study, two spray-dried formulations of the antibiotic nitrofurantoin were prepared using 2FN and 3FN by spray-drying a solution of 1:1 *w*/*w* mixture of NF with HPMC. The dry powder formulations were characterised in terms of their Physicochemical properties, aerosolization performance and finally, their efficacy was tested in vitro to determine their potential as a novel treatment for emphysema.

HPMC (viscosity 80–120 cp, 2% in H_2_O) was chosen as the excipient in these formulations due to the utility of this polymer in the development of spray-dried formulations. HPMC is a semisynthetic, biocompatible polymeric excipient most commonly used in oral formulations, however, HPMC has been shown to prolong drug release and improve the aerosolisation of inhaled formulations [20]. Furthermore, the polymer has been consistently found to be non-toxic and inert when inhaled [19] with a posited no-observed-adverse-effect level (NOAEL) of 5 mg/kg/day in humans when ingested orally [21]. Additionally, HPMC has been previously used as an excipient for spray drying nitrofurantoin and was found to improve the bioavailability of the resulting particles. However, the particle size of this formulation was 125–160 µm, implying that the powder was not intended for use as an inhaled therapy [31]. HPMC was chosen to be spray-dried with nitrofurantoin as the polymer has not been found to pose health risks to humans, improves the physicochemical characteristics of spray-dried formulations and has been extensively studied.

The choice of nozzle resulted in significant differences in the Physico-chemical characteristics between the 2FN and 3FN formulations. These altered characteristics were due to differences in feed preparation and nozzle geometry mediated mechanisms of particle formation, potentially changing drug distribution patterns within the particles, intrinsic drug solubility and dissolution rates, thus altering the therapeutic efficacy of NF [12,13,14,15]. Spray-drying using 2FN requires a mixture of a drug (NF) and excipient (HPMC) fed into the 2FN nozzle via a single channel, resulting in particles comprised of NF dispersed throughout HPMC. While this conventional approach has been popular, many studies have shown that the drugs are distributed uncontrollably throughout the surface and core of the particles, potentially leading to rapid drug release [14,16,17]. The 2FN method of spray drying was also limited by the precipitation of nitrofuranotin after extended periods of spray drying (>5 h), requiring the production of 2FN to be limited to approximately 4.5 h or smaller batches of the formulations. The drug content of both 2FN (42%) and 3FN (34.3%) was found to be lower than the theoretical 50%, potentially due to drug being lost during the spray drying process or possibly due to inconsistencies in the HPMC content of atomised droplets. In contrast to 2FN-mediated spray drying, the 3FN employs two different channels to feed NF and HPMC separately. The simultaneous feeding of NF by the 3FN inner channel allows NF to be encapsulated by HPMC fed from the outer channel, producing single droplets during atomization and therefore altering the characteristics of the resulting particles. However, the use of separate channels during 3FN spray drying may negatively impact the homogeneity of the resulting particles. HPMC based formulations have been shown to typically produce superior drug content with greater ratios of HPMC to drug [32], suggesting that spray drying may select for particles with higher ratios of HPMC to nitrofurantoin. Selecting for particles with a greater ratio of HPMC to nitrofurantoin would further reduce the drug content of 3FN powder given the wider variability in atomised droplets produced by a 3FN. The encapsulation of NF within a shell of the excipient HPMC is likely to mask the hydrophobicity of the drug, enhance the solubility and allow the drug to release in a slower and sustained fashion [33,34].

Dissolution studies revealed the significantly higher release of the NF from 2FN powders as compared to 3FN over three hours (Figure 4). The Korsmeyer-Peppas ‘Power Law’ model of drug dissolution was shown to best model the dissolution of both 2FN and 3FN, indicating that dissolution of both powders is governed by HPMC. The power law model of dissolution describes the release of a drug from a polymeric system, such as the HPMC matrix created in 2FN and 3FN formulations [35,36]. Additionally, the release exponent (N-values) of 2FN and 3FN was found to be 0.636 and 0.661, respectively, indicating that non-Fickian diffusion is occurring in both formulations [30]. This mechanism of diffusion implies that moisture causes the particles to swell at the surface, forming a distinct swollen phase and a dry, glassy phase in each particle as the moisture penetrates inward [37,38]. This border between the swollen phase and the glassy phase disrupts the passage of moisture into the particle, favouring drug diffusion near the particle surface. This diffusion mechanism indicates that particles with surface-enriched NF would demonstrate a greater rate of dissolution. The similarity of the dissolution curves was then assessed by calculating the similarity factor (*f*2). *f*2 was determined to be 50.7, potentially indicating that the curves are similar (50–100). However, a result of 50.7 was deemed inconclusive as it closely borders on different (0–50). As an alternative, the difference factor (*f*1) was calculated to clarify the similarity of the two curves, resulting in a value of 23.91 that indicated that the two curves are different (15–100). The discrepancy between the *f*1 and *f*2 values was due to *f*1 being proportional to the average difference between dissolution curves, whereas *f*2 is inversely proportional to the average squared difference between dissolution curves [39]. The difference in dissolution profiles could be attributed to the localisation of NF throughout the 2FN particles and within 3FN particles. A portion of NF has been distributed on the surface of 2FN particles, enabling faster NF dissolution and indicating potential as an immediate release therapy. Conversely, the 3FN formulation consisted of drug particles encapsulated within a coating of HPMC and displayed a sustained, slow release of the NF. These results were consistent with literature about the dissolution rates of 2FN and 3FN-derived formulations [14].

Other physicochemical characteristics of the powder were also found to contribute to the slower dissolution rate of 3FN, such as the X-ray diffraction results of the two formulations. 2FN was found not to display intense crystalline peaks indicative of raw nitrofurantoin at 2θ 16.8 and 33.5°, whereas 3FN displayed inferior crystalline peaks at 2θ 16.8 and 33.5°, implying that 3FN remains partially crystalline, whereas spray-dried 2FN formulation was found to be highly amorphous (Figure 2). The change in crystalline peaks implies that both formulations were more amorphous after spray drying, however, the 3FN formulation had maintained some crystallinity compared to 2FN formulation. This result aligns with scientific literature surrounding 2FN and 3FN nozzles as 3FN has previously been found to produce more crystalline particles than 2FN [14,40]. The crystallinity findings determined by XRD did not correlate with the thermal properties of 2FN and 3FN, as determined by DSC and TGA (Figure 3), however, XRD is considered a more robust and accurate method of determining crystallinity [41,42]. Notably, amorphous solids are known to have higher Gibbs free energy than crystalline solids, resulting in greater solubility of amorphous drugs compared to crystalline drugs [43,44].

Furthermore, 3FN particles were found to have significantly larger particle sizes compared to 2FN formulation (Table 2), further impeding dissolution due to the lower surface area to volume ratio of larger particles [45]. Particle size has also been shown to influence the aerosolization and dissolution properties of orally inhaled drugs [46]. The optimum particle size for inhaled therapies is recommended to be within the range of 2–5 µm, and thus likely to deposit in the lower region of alveoli and bronchi [47]. The size differential between 2FN and 3FN formulations is most likely due to the spray drying process as the exterior localisation of hydrophilic HPMC in 3FN would cause the particle to retain water, slowing the drying process and thereby preventing 3FN particles from shrinking as much during the drying process. Conversely, the consistent dispersion of NF throughout 2FN reduces the particle water retention, allowing the particle to dry more quickly. Both formulations showed that the majority of the powders were less than (50% of the particle population) of 5 µm (Table 2). Additionally, 2FN displayed a significantly lower particle size compared to 3FN, suggesting that 2FN would penetrate further to the lower regions of the lung. NGI was used to verify the aerosolization performance of 2FN and 3FN. Drug distribution analysis revealed that NF was primarily deposited between stages S3–S6 for 2FN whereas 3FN is primarily deposited between stages S2–S4. The differences in deposition profiles produced are related to the characteristics such as particle size, morphology and density [46]. The 2FN was found to have significantly higher FPF% compared to 3FN, confirming that 2FN can penetrate deep regions of the lung. In addition, the MMAD of 3FN is significantly higher compared to that of 2FN, aligning with the previous finding that 3FN particle size is greater than that of 2FN. Notably, powders with density in the range of 0.1–0.4 g/cm^3^ show better aerosolization, flowability and enhanced deposition at the lower lung region [14]. Both powders showed tapped density within this range, however, 3FN powders displayed a larger tapped density, suggesting that the difference in the aerosolization behaviour of the powders is related to the higher particle size and density of 3FN. The higher density of 3FN could be attributed to the formation of more hydrophilic 3FN particles, resulting in more hygroscopicity.

The particle formation process gave rise to a corrugated morphology for both formulations despite the difference in theoretical NF localisation. The ratio of the solvent evaporation rate to solute diffusivity is described as a Peclet number [48,49]. In the case of both 2FN and 3FN, the corrugated particle morphology suggests a high Peclet number, as the solvent evaporation rate of the acetone-water mixture exceeded the diffusivity of the solute, NF. A higher solvent evaporation rate prevents solute from remaining consistently dispersed as atomised droplets of feed solution dry, resulting in the formation of a dense outer shell phase surrounding a hollow interior phase. The outer shell of the 2FN formulation deforms to fill the interior void, resulting in a ‘crumpled’ or corrugated particle morphology. HPMC has been previously used in spray drying with high Peclet number formulations to ensure that the polymeric outer shell deforms elastically, rather than resulting in burst particles [50]. The corrugated morphology can be beneficial to formulations as the outer shell formation enriches the NF surface concentration of 2FN particles, increasing the rate of dissolution. Notably, the hypothesised interior localisation of NF during 3FN spray-drying would result in reduced NF surface enrichment. Furthermore, a crumpled particle morphology also provides the benefit of superior dispersibility as the troughs and dimples between folds minimise the contact area of the particle [48]. The 3FN formulation produced highly corrugated particles, however, the exterior coating of water and HPMC would result in a lower solvent evaporation rate than the acetone-water mixture used in 2FN and thus, a lower Peclet number.

Moisture sorption analysis revealed that raw NF, 2FN, 3FN and raw HPMC were all hygroscopic, though raw NF was far less hygroscopic (Figure 4). Notably, the sorption profiles of raw HPMC and raw NF were consistent with the literature [51,52]. 3FN and raw HPMC were found to have reversible moisture sorption profiles, whereas the 2FN sorption profile was only reversible after the first sorption cycle. The difference in the result of the two formulations can be attributed to the high surface concentration of HPMC in the 3FN formulation, causing the 3FN sorption profile to display a highly similar pattern to that of raw HPMC. Notably, the change in mass of 2FN is sharply reduced at 60% RH. This sudden decrease can likely be attributed to the elimination of residual solvents and the recrystallisation of the amorphous formulation as the 2FN sorption profile was found to be reversible after the first cycle. The reversibility of the 3FN sorption profile indicates that the 3FN formulation has superior stability to 2FN. Furthermore, moisture content analysis revealed a higher moisture content in the powder produced by 3FN which could be attributed to the particle surface being dominated by the hygroscopic HPMC polymer [52].

A transport study was performed to investigate the ability of the two spray-dried formulations to cross the epithelial layers of a human alveolar cell line using an air-liquid interface model (Figure 7). The cumulative mass of NF transported after 6 h for 2FN formulation was significantly higher (2.9 µg) in comparison to 3FN (1.8 µg). The higher transport of 2FN can be attributed to differences in particle characteristics such as greater dissolution, smaller particle size and amorphous nature of 2FN when compared to 3FN. Notably, a negligible amount of nitrofurantoin (~1%) was found inside the cells for both 2FN and 3FN after the 6h transport study, suggesting that the NF spray-dried particles were transported via the paracellular tight junctions. These results correlate with the findings of a previous study that showed NF to use a paracellular route of transport [53]; however, further studies are required to determine the mechanism of NF drug transport.

Finally, an in vitro efficacy study was performed to compare the efficacy of 2FN or 3FN on fibroblast ECM protein deposition after exposure and subsequent transport of NF across the epithelium (Figure 8). The ECM proteins fibronectin, periostin, tenacin-C, Collagen-IV and perlecan were selected as they cover a range of functions within the ECM, such as structural proteins (collagen-IV), signalling proteins (perlecan) and proteins relating to dynamic cellular activity such as adhesion and differentiation (fibronectin, tenacin-C, periostin) [54,55,56,57]. Both 2FN and 3FN formulations demonstrated a significant increase in collagen-IV and periostin compared to control (untreated cells). Importantly, 2FN showed a significantly higher collagen-IV production compared to 3FN, highlighting the better efficacy of the 2FN formulation compared to 3FN. This significant increase in collagen-IV production by 2FN treated cells is most likely due to the superior transport rate of 2FN resulting in a higher concentration of NF, and thus producing a greater collagen-IV upregulation. The upregulation of collagen-IV may be beneficial to the emphysematous lung as it is a major protein in the lung basement membrane, providing essential physical structure to the lung [58]. The exact relationship between collagen-IV and emphysema remains unclear, however, collagen-IV turnover is significantly higher during COPD and predictive of mortality and lung function [58,59,60], suggesting that the protein plays a role in emphysema progression. Likewise, the role of periostin in emphysema is not yet fully understood. Notably, no correlation was found between disease progression and plasma periostin levels [61], however, plasma periostin was found to significantly correlate with the improved lung function (measured by FEV1) of emphysema patients undergoing treatment of inhaled corticosteroids and long-acting beta antagonists [62]. The link between the proteins upregulated by 2FN or 3FN and emphysema remains unclear, therefore, further exploration of 2FN and 3FN efficacy is necessary to determine the future potential of the formulations as a treatment for emphysema. Similarly, the toxicity of 2FN and 3FN should be studied further to ensure that the therapeutic effect outweighs any potential harm.

This study aimed to develop and compare spray-dried formulations prepared using a 2FN and a 3FN as potential inhaled therapies for emphysema. Comparatively, the 2FN formulation was found to have superior yield, particle morphology, deposition profile, and drug release. Furthermore, 2FN was found to induce a greater upregulation of the ECM proteins collagen-IV, suggesting a potentially superior therapeutic effect.

## Figures and Tables

**Table 1:** Physicochemical properties of spray-dried powders (Mean ± SD, *n* = 3, * *p* < 0.05).

	Powder Yield (%)	Density (g/cm^3^)		Moisture Content (%)	Drug Content (%)
		Bulk	Tapped		
2-Fluid Nozzle (2FN)	54.49	0.11 ± 0.002	0.14 ± 0.005	2.5 ± 0.6	42.0 ± 1.9
3-Fluid Nozzle (3FN)	29.56	0.14 ± 0.005 *	0.19 ± 0.02 *	3.1 ± 0.4 *	34.3 ± 2.7 *

**Table 2:** Particle size and span range of 2FN and 3FN as measured by laser diffraction (Mean ± SD, *n* = 3, Unpaired Student’s *t*-test, * *p* < 0.01).

	2-Fluid Nozzle	3-Fluid Nozzle
D_10_ (μm)	0.60 ± 0.02	0.42 ± 0.04 *
D_50_ (μm)	1.28 ± 0.04	2.92 ± 0.08 *
D_90_ (μm)	4.24 ± 1.88	9.67 ± 2.5 *
Span	2.82 ± 1.3	3.16 ± 0.8

**Table 3:** Aerodynamic profiles of 2FN and 3FN as assessed by next-generation impactor (NGI). Fine particle fraction (FPF), median mass aerodynamic diameter (MMAD), geometric standard deviation (GSD) an emitted dose (ED) were determined from resulting NGI data (Unpaired Student *t*-test, Mean ± SD, *n* = 3, * *p* < 0.05).

	2FN	3FN
FPF (%)	87.38 ± 2.8	40.03 ± 5.8 *
MMAD (μm)	1.78 ± 0.05	4.36 ± 0.39 *
GSD	1.74 ± 0.07	1.61 ± 0.04
ED (mg)	6.90 ± 0.78	7.32 ± 0.12

**Table 4:** Nitrofurantoin dissolution profile modelling and comparison of 2FN and 3FN (difference (*f*1) and similarity (*f*2)).

	Difference (*f*1)	Similarity (*f*2)	Zero Order (R^2^ Value)	First Order (R^2^ Value)	Higuchi (R^2^ Value)	Korsmeyer-Peppas (R^2^ Value)	Hixson-Crowell (R^2^ Value)
2FN	23.91	50.71	0.8717	0.9757	0.9622	0.9882	0.9527
3FN			0.8656	0.9493	0.9687	0.9916	0.9272

## Data Availability

Not applicable.
